# Charge-driven antiviral responses enhance autoreactivity in severe COVID-19

**DOI:** 10.21203/rs.3.rs-10285086/v1

**Published:** 2026-07-25

**Authors:** Renren Wen, Nathan Witman, Daniel Villalobos-Garcia, Mei Yu, Archana Molangiri, Areeb Bajwa, Mary Beth Graham, Shawn Jobe, Weiguo Cui, Jieqing Zhu, Demin Wang

**Affiliations:** Versiti / Blood Research Institute; Medical College of Wisconsin; Versiti Blood Research Institute; Versiti / Blood Research Institute; Versiti Blood Research Institute; Versiti Blood Research Institute; Medical College of Wisconsin; Versiti Blood Research Institute; Northwestern University Feinberg School of Medicine; Blood Research Institute; Bloodcenter of Wisconsin

## Abstract

Viral infections are often accompanied by the emergence of antibodies that recognize both viral and self-antigens, a phenomenon largely attributed to molecular mimicry. However, this mechanism does not readily explain the emergence of antiviral antibodies with broad autoreactivity and polyreactivity toward structurally unrelated antigens during acute infection. Here, we demonstrate a charge-driven mechanism underlying the acquisition of autoreactivity by antiviral antibodies. Using BCR sequencing and recombinant antibody cloning from B cells of COVID-19 patients, we found that antiviral antibodies gained autoreactivity through enrichment of positively charged residues in complementarity-determining regions (CDRs). Germline reversion showed that increased spike protein reactivity (S2 domain and RBD) was associated with net positive charge gains in heavy- and light-chain CDRs. Critically, all the positively charged antibodies that gained reactivity to the negatively charged S2 subunit simultaneously increased autoreactivity. Docking and charge mutagenesis of interacting residues confirmed that positive charge is critical for both spike and autoantigen binding. At the population level, antibody repertoires against the more negatively charged Wuhan SARS-CoV-2 spike variant had significantly higher positive charge than those reacting to Omicron variants. Consistently, the charge of hemagglutinin variants and differing subunits drove similar repertoire differences in influenza. Together, these findings identify charge-driven antiviral affinity maturation as a previously unrecognized mechanism linking antiviral immunity to autoreactive antibody generation.

## Introduction

Infectious diseases have long been associated with the induction of autoreactive antibodies, yet the mechanisms by which antiviral immune responses give rise to autoreactivity remain incompletely understood^1–4^. Multiple models have been proposed, including molecular mimicry, bystander activation, and epitope spreading, but these models have yet to fully explain the frequent emergence of antibodies that recognize both viral and autoantigens during acute infection, often with broad autoreactive or polyreactive binding properties^5–8^. Polyreactive antibodies, which react to a variety of structurally distinct antigens with varying affinities, have been documented across viral, bacterial, and parasitic infections^2–4,9–11^. Although infection-associated autoreactive antibodies are often transient, their persistence in some settings has been linked to chronic immune dysregulation and persistent autoimmune disease, underscoring the need to understand the origin of the autoreactive antibody responses^12,13^.

COVID-19 is characterized by profound immune activation and is frequently accompanied by the induction of autoantibodies, including anti-nuclear antibodies (ANA)^8,14,15^. ANAs have long been identified as a key pathological agent in systemic lupus erythematosus (SLE) and has more recently been associated with increased COVID-19 and influenza disease severity^8,16–18^. ANA antibodies are often enriched for positively charged residues that facilitate recognition of highly charged antigens, including histone/DNA complexes, double stranded DNA, and ribonucleoproteins. However, what causes the generation of ANA antibodies in infectious diseases remains poorly understood. Recent studies, including work from our laboratory, have shown that antibodies targeting the SARS-CoV-2 spike protein, particularly the receptor-binding domain (RBD), can also react to platelet factor 4 (PF4) complexed with polyanions, as well as with a range of other structurally diverse autoantigens, including those detected by ANA assays^8,19^.

Here, we utilize COVID-19 as a model to understand the origin, specificity, and binding properties of autoreactive antibodies arising in viral infection. By integrating clinical associations, single-cell V(D)J sequencing, germline reversion, recombinant antibody analysis, and population-level repertoire analysis, we show that germline-reverted SARS-CoV-2 spike-reactive antibodies gain positive charge in antibody paratopes as they enhance both viral and autoreactivity. We found that this autoreactivity was associated with a gain in reactivity towards the S2 and RBD domain of the spike protein. Importantly, all antibodies that gained S2 reactivity, the most negatively charged subunit of spike, demonstrated enhanced autoreactivity. Moreover, we found that antibody repertoires recognizing the ancestral Wuhan strain were enriched for positive charges in the heavy-chain complementarity-determining region 3 (HCDR) compared with those recognizing the Omicron variant, consistent with reports that the Omicron spike has a more positive surface charge than the Wuhan strain^20^. Further, we also determined that the hemagglutinin protein of the influenza virus generates differentially charged repertoires based on the charge of the variant and subunit. Together, our work identifies a novel mechanism by which charged viral epitopes drive affinity maturation that enhances autoreactivity in viral infections.

## Results

### Elevated ANA responses track with measures of COVID-19 disease severity and respiratory failure.

**Figure 1:**
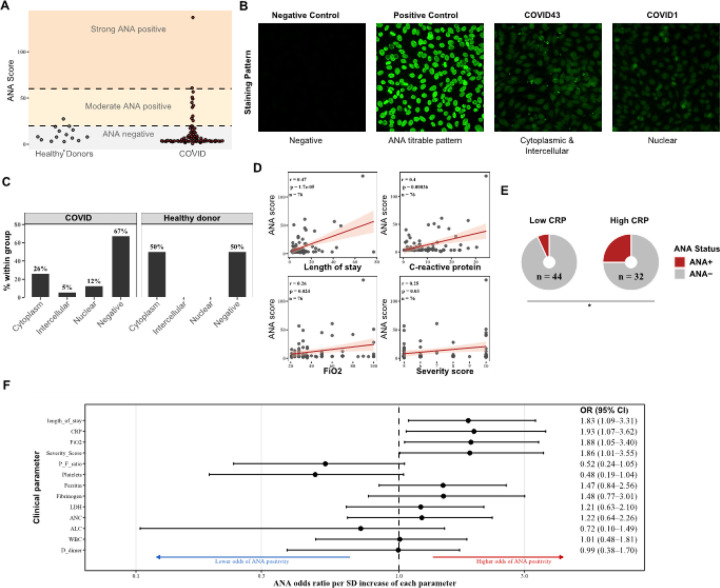
Autoreactive antibodies in hospitalized COVID-19 patients are associated with disease severity and inflammation. (**A**) Anti-nuclear antibody ELISA results for COVID-19 patients (COVID) (n = 76) and healthy donors (n= 14). Thresholds were drawn at ≥ 20 ANA score as moderate and > 60 as strong positive. (**B**) Representative HEp-2 immunofluorescence staining patterns using sera from ANA-negative control, ANA-positive control, and COVID-19 patients. Shown are examples of ANA titratable pattern, cytoplasmic & intercellular, and nuclear staining patterns observed among ANA-positive COVID-19 plasma. (**C**) Distribution of HEp-2 staining patterns among ANA-positive COVID-19 patients (n = 75) and healthy donors (n = 7) plasma IgG. Bars indicate the percentage of patients exhibiting cytoplasmic, mitochondrial, nuclear, nucleolar, or negative staining patterns. Patients can belong to multiple categories depending on the presented staining patterns. (**D**) Correlation of plasma ANA scores with clinical measures of disease severity in hospitalized COVID-19 patients. Pearson correlation coefficients, p values, and sample sizes are shown. (**E**) Comparison of ANA status in patients with low versus high C-reactive protein (CRP). Patients were stratified by CRP concentration (≤10 mg/L versus >10 mg/L), and ANA status (ANA+ = ANA score > 20; ANA− = ANA score < 20). Statistical significance was assessed by Fisher’s exact test. *p < 0.05. (**F**) Associations between clinical parameters and ANA positivity were assessed using univariate logistic regression. Clinical variables were standardized (z-scored), and odds ratios represent the change in odds of ANA positivity (ANA score > 20) per 1 standard deviation increase in each parameter. Points indicate odds ratios, and horizontal lines represent 95% confidence intervals. The dashed vertical line denotes no association (odds ratio = 1).

Previous work has shown that ANA titers increase with disease severity in COVID-19^8^. To further characterize the clinical features associated with ANA in COVID-19 patients, we first confirmed the development of ANA antibodies within our cohort via ANA ELISAs on patient plasma. Indeed, strong ANA scores (> 60) were identified within COVID-19 patients, while many patients fell within the moderately positive range (≥ 20; < 60 ANA score) (Fig. 1A). We also examined non-COVID patients with acute respiratory symptoms (ARS) and healthy donors post vaccination. Surprisingly, ~ 30% (6/20) of non-COVID ARS patients and ~ 40% (2/5) of our limited vaccinee cohort were ANA positive, suggesting immune responses beyond acute SARS-CoV-2 infection may also contribute to ANA development (Supplemental Fig. 1A). To understand the antigenic targets of ANA antibodies in COVID-19 patients, we characterized antibody reactivities in patient plasma by performing immunofluorescence on HEp-2 slides and identifying staining patterns based on the CDC's definition of autoantibody immunofluorescence staining patterns^21^. Among ANA-positive sera, the majority of patients exhibited cytoplasmic and nuclear targeted antibodies, consistent with bona fide ANA antibodies (Fig. 1, B and C). These patterns are consistent with reactivities for dsDNA, histones, nucleosomes, and other canonical autoantigens^21^. These data demonstrate that ANA antibodies that develop in COVID-19 target canonical autoantigens and confirm ANA development in our cohort.

To identify the clinical parameters associated with ANA positivity, we performed correlation and odds ratio analyses. We found moderate correlations with length of stay (r = 0.47) and C-reactive protein (CRP) (r = 0.4), as well as weak but significant correlations with supplemental oxygen requirement (FiO2) (r = 0.26) and severity score (r = 0.25), (Fig. 1D). Comparison of patients with low (≤ 10 mg/L) versus high (> 10 mg/L) CRP demonstrated a significant enrichment of ANA positivity among patients with elevated CRP (Fig. 1E), consistent with previous findings^8^. Similar trends were observed when we compared both CRP and severity score between patient groups and healthy donors (Supplemental Fig. 1, B and C). Because ANA scores of the majority of patients were below the positivity threshold (Fig. 1A), we examined whether patients in the upper quadrant of each clinical parameter distribution were more likely (higher odds) to be ANA-positive (positive vs negative, defined as ≥ 20 ANA score). Elevated CRP and FiO2 values were associated with increased odds (> 4x more likely) of ANA positivity, whereas higher oxygenation efficiency (P_F_ratio) was associated with lower odds (< 0.5x less likely) (Fig. 1F). Taken together, these data show that patients with high inflammation and respiratory distress are at greater risk of developing autoreactive antibodies.

### Polyreactivity to self and viral antigens is associated with a charged HCDR3 motif.

To investigate immunologic features underlying the emergence of autoreactive antibodies during severe COVID-19, we used single-cell V(D)J sequences from B cells of 11 hospitalized patients reported in our previous publication and cloned 200 recombinant antibodies from paired heavy- and light-chain sequences^22^. Because antigenic drivers of autoreactive responses during severe COVID-19 remain poorly defined, V(D)J sequences were not cloned from antigen-sorted B cells, but were selected based on two criteria: evidence of clonal expansion (≥ 3 cells sharing the same V(D)J rearrangement and > 85% HCDR3 sequence homology) or the presence of an RKH/Y5 motif, a sequence feature previously associated with autoreactive platelet-activating antibodies in COVID-19 and heparin-induced thrombocytopenia^19,23^.

**Figure 2:**
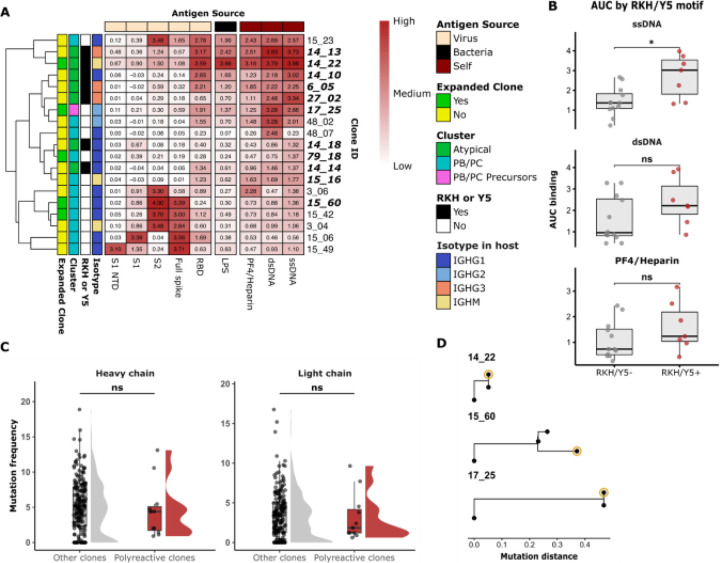
Autoreactive antibodies exhibit antiviral reactivity and motif enrichment. (**A**) Heatmap summarizing antigen reactivity profiles of cloned antibodies. Antibodies are hierarchically clustered based on area under the curve (AUC) values from ELISA assays against viral antigens (SARS-CoV-2 full spike and spike subdomains), bacterial antigens, and self-antigens. Annotation bars indicate HCDR3 amino acid length, presence of the RKH/Y5 motif, isotype, clonal expansion status, and inferred B cell subset identity based on previously published single-cell annotations. Color intensity reflects AUC binding within each antigen column (high to low). Bolded Clone IDs indicate the 11 polyreactive clones selected for downstream analysis. (**B**) Autoantigen AUC binding comparisons between antibodies with (RKH/Y+)(n = 7) and without (RKH/Y5-)(n = 12) the RKH/Y5 motif. (**C**) Comparison of immunoglobulin heavy-chain and light-chain mutation frequencies between polyreactive antibodies (n = 11) and other cloned antibodies (n =189). Colored peaks parallel to boxplots indicate dot distribution for each group. (**D**) Representative clonal lineage trees of polyreactive antibody clones reconstructed using IgPhyML. Lineage trees depict inferred relationships from germline sequences. Scale bars indicate nucleotide substitutions per site. The yellow circle indicates the clonal sequence tested in the ELISAs in panel A. Shown are representative clones from different annotated clusters (14_22, 15_60, and 17_25). Statistical analyses were performed using Wilcoxon rank-sum test. *p < 0.05.

We first screened transfection supernatants for binding to viral antigens, identified reactive clones, and rescreened concentrated IgG (100ug/mL) for full-length spike, spike subunits, and canonical autoantigens (dsDNA, ssDNA, and PF4/heparin complexes). We identified 19 of 200 antibodies from 7 of the 11 patients as the most reactive, defined as those within the top ~ 10% of mean OD450 values across all antigens (Supplemental Table 1). The 19 antibodies were then subjected to serial dilution ELISAs to quantify binding breadth across a panel of viral, bacterial, and autoantigens, including dsDNA, ssDNA, and LPS (Fig. 2A). Area under the curve (AUC) analysis revealed that a substantial fraction of antibodies with viral reactivity also bound multiple autoantigens. Of the 19 antibodies, 36% contained the RKH/Y5 motif and had strong autoreactivity (Fig. 2A). Indeed, comparison of AUC for autoantigens revealed significant increases in ssDNA reactivity within antibodies that contained this motif (Fig. 2B). Although other autoantigen reactivities also appeared to be increased, these were not significant, likely due to the limited clone number (Fig. 2B). This is consistent with previous observations that increased cationic and tyrosine residues are associated with antibody auto- and polyreactivity^24–26^. Thus, we selected 11 of the 19 antibodies with both viral and autoreactivity for downstream analysis, and they are hereafter referred to as polyreactive antibodies.

We next examined whether polyreactivity could be explained by differences in somatic hypermutation. Comparison of mutation frequencies across total heavy- and light-chain sequences revealed no significant differences between the polyreactive antibodies and the other 189 cloned antibodies (Fig. 2C), indicating that autoreactivity is unlikely to be driven by mutational burden. Finally, to explore the evolutionary trajectories of polyreactive clones, we reconstructed clonal lineage trees for 3 of the 5 expanded clones in our polyreactive antibody cohort^22^. Clonal trees were constructed based on inferred germline sequences and mutational divergence of expanded B cell clones (Fig. 2D). Clones 15_60 and 17_25 exhibited reactivity to both viral and autoantigens, with autoreactivity occurring despite having higher mutation frequencies than 14_22 (Fig. 2D). Therefore, consistent with the overall mutational burden analysis, lineage analysis showed that polyreactivity did not appear to depend on overall mutational burden or reactivity for viral and autoantigens, in contrast to current perceptions that low mutational burden yields more autoreactivity. Together, these data suggest that polyreactive antibodies that recognize both viral and autoantigens are not influenced solely by the mutational burden but rather have notable enrichment for the positively charged RKH/Y5 motif.

### Polyreactive antibodies converge functionally without sequence or paratope convergence.

**Figure 3:**
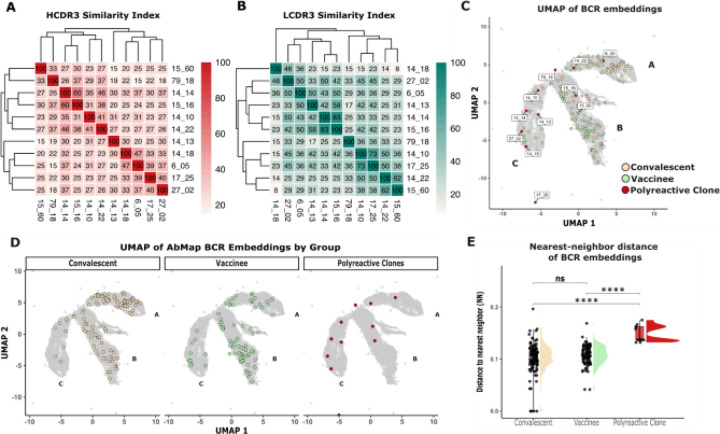
Polyreactive antibody reactivity is independent of sequence homology and paratope convergence. (**A**) Pairwise heavy-chain CDR3 (HCDR3) sequence similarity among polyreactive antibodies (n = 11). Heatmap displays percent amino acid identity between HCDR3 sequences, with hierarchical similarity clustering. Values indicate percent similarity. (**B**) Pairwise light-chain CDR3 (LCDR3) sequence similarity among polyreactive antibodies (n = 11). Heatmap displays percent amino acid identity with hierarchical similarity clustering. (**C**) Paratope embedding analysis of antibody heavy-chain repertoires using AbMAP. UMAP of predicted heavy-chain paratope features is shown for polyreactive antibodies cloned in this study (red) (n = 11), alongside SARS-CoV-2 RBD–specific antibodies derived from convalescent individuals (tan) (n = 105) and vaccinated individuals (green) (n = 79) obtained from previously published datasets. UMAP structure was generated utilizing 24,074 heavy-chain sequences from our cohort single-cell V(D)J-sequenced B cells. UMAP lobes are labeled as A, B, and C. (**D**) UMAP split by patient subsets, and (**E**) quantified nearest-nearest neighbor calculations showing clustering of similar heavy-chain paratopes within each group on the UMAP. For each heavy-chain sequence, Euclidean distance to its closest neighbor was calculated. Statistics were calculated using Wilcoxon rank-sum test. ****p < 0.0001.

Given the enrichment of RKH/Y5 motifs among polyreactive antibodies, we first asked whether polyreactivity was driven by sequence convergence within the critical antigen-binding regions. To assess this, we compared HCDR3 and light-chain CDR3 (LCDR3) sequences of the polyreactive antibodies using pairwise similarity analysis and visualized percent sequence identity as a clustered heatmap. Across the polyreactive antibodies, no HCDR3 sequences shared greater than 60% sequence identity with any other clone (Fig. 3A). LCDR3 similarity analysis revealed a single larger cluster alongside another smaller ones (Fig. 3B). However, these clusters did not correspond to shared antigen-binding profiles, reflecting more limited light-chain diversity and consistent with prior work suggesting that light-chain sequence similarity alone is a poor predictor of antibody reactivity^27^. Together, these analyses indicate that polyreactive binding across similar antigens arises independently of sequence homology in either HCDR3 or LCDR3 regions.

Because antibody CDR3 sequence diversity does not necessarily recapitulate paratope diversity or the structural properties of the CDRs, we next assessed antibody paratope similarity using AbMAP, a machine-learning pipeline that generates antibody similarity embeddings based on predicted paratope structure and physicochemical features derived from established protein-language models^28^. To both validate our pipeline and compare other antiviral immune responses to our dataset, we integrated our hospitalized COVID-19 antibody dataset with previously published RBD-specific antibodies derived from vaccinated individuals and convalescent patients to generate a paratope-similarity UMAP using AbMAP^29,30^. The UMAP revealed that RBD-specific antibodies from vaccinated and convalescent cohorts clustered within lobes A and B of the UMAP (Fig. 3, C and D), consistent with converging paratopes to a few specific viral epitopes^29,31^. In contrast, polyreactive antibodies distributed broadly across all three lobes, exhibiting minimal co-clustering despite shared antigen reactivity (Fig. 3, C and D). To quantitatively assess paratope relationships, we calculated nearest neighbor (NN) distances, the UMAP distance from each clone to its closest neighbor within each group. In the NN analysis, greater distances between clones indicates greater differences in predicted paratope structure. NN analysis confirmed that paratopes from both convalescent patients and vaccinees clustered significantly closer to one another than those of polyreactive clones. (Fig. 3E). These data suggest the overall paratope diversity within polyreactive clones is greater than that of antigen-sorted anti-spike antibodies. Together, these findings demonstrate that polyreactive antibodies do not arise through sequence or paratope convergence, which is inconsistent with conventional molecular mimicry and suggests other mechanism of polyreactivity.

### Polyreactivity is mediated by charge-dependent antigen recognition.

**Figure 4:**
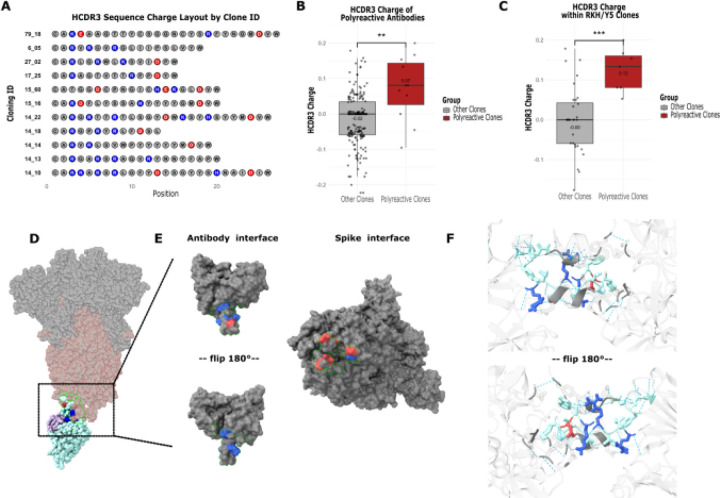
Positively charged paratopes underly polyreactive antibody binding. (**A**) Amino acid composition of heavy-chain CDR3 (HCDR3) regions from polyreactive antibodies. Each row represents an individual clone, with residues plotted by position along the HCDR3. Positively charged residues (arginine, histidine, and lysine) are blue, and negatively charged residues (aspartic acid and glutamic acid) are red. (**B**) Comparison of HCDR3 charge between polyreactive antibodies (n = 11) and other cloned antibodies (n =189). Box labels indicate mean; points represent individual antibodies. Statistical significance was assessed using a Wilcoxon rank-sum test. (**C**) HCDR3 charge comparisons of polyreactive antibodies (n = 7) and other cloned antibodies (n = 32) within antibodies containing the RKH/Y5 motif. Statistical significance was assessed using a Wilcoxon rank-sum test. (**D**) Predicted interaction interface between polyreactive antibody clone 14_22 and the SARS-CoV-2 spike protein. Gray and salmon colors represent the S1 and S2 domains of the spike protein, respectively. The interface region is outlined in green. Cyan and magenta represent the heavy and light chains of the antibody Fab, with charged residues within the interface highlighted in blue for positive and red for negative. (**E**) Antibody and spike interface residues. The interface region is outlined in green, with charged amino acids colored in blue for positive and red for negative. (**F**) Ribbons model of 14_22/spike interface. Lines indicate hydrogen bonds between side chains. Dark gray lines represent portions of the spike in the interface, and cyan lines represent regions of the HCDR3 within the interface. **p < 0.01, ***p < 0.001.

Previous studies in SLE demonstrated that increased positive charge within CDRs can promote autoreactivity and polyreactivity, particularly to DNA^24^. Thus, we investigated whether shared charge features underlie the reactivities of the polyreactive antibodies. We first characterized the distribution of charged residues within the HCDR3 and LCDR3 regions of the 11 polyreactive antibodies and found that the HCDR3 appeared enriched for positively charged amino acids (Fig. 4A and Supplemental Fig. 2A). We next compared HCDR3 charge between the 11 polyreactive antibodies and the other 189 cloned antibodies and found significantly greater net positive charge in the polyreactive antibodies (Fig. 4B)^24^. To control for the inherent enrichment of positively charged residues introduced by our sequence selection strategy, we then restricted this analysis to antibodies containing the RKH/Y5 motif. Even within the RKH/Y5 containing subset, polyreactive antibodies retained a significantly higher HCDR3 positive charge than nonpolyreactive clones (Fig. 4C), indicating that as positive charge increases, so does reactivity to viral and self-antigens.

To explore how charge enrichment might facilitate antibody binding, we performed computational docking analyses using the Fabs of 3 polyreactive antibodies, with clone 14_22 as a representative example. We generated predicted Fab structures for these antibodies and then unbiasedly docked them to the crystallized structure of the Wuhan spike protein. Clone 14_22 was then visualized as a representative sample using ChimeraX. The 14_22 Fab was predicted to dock in the highly negative patch of the S2 domain, right before the stalk domain that anchors spike to the viral membrane (Fig. 4D and Supplemental Fig. 2B). Additionally, interface analysis of both the 14_22 Fab and spike protein showed a high dependency for positively charged amino acids for antibody docking (Fig. 4, E and F). To determine if this was consistent across the other polyreactive antibodies, all 3 docked complexes were analyzed using PDBePISA to characterize interface composition and interaction types. In the predicted Fab–spike complexes, all interfaces relied heavily on charged interactions through salt bridges in the heavy-chain (Table 1), consistent with positive charge contributing to the observed polyreactivity. While docking analyses are predictive in nature, these data support the model that positive charge is the primary driver of reactivity to negatively charged viral epitopes.

### Negatively charged S2-directed binding drives reactivity to autoantigens.

**Figure 5:**
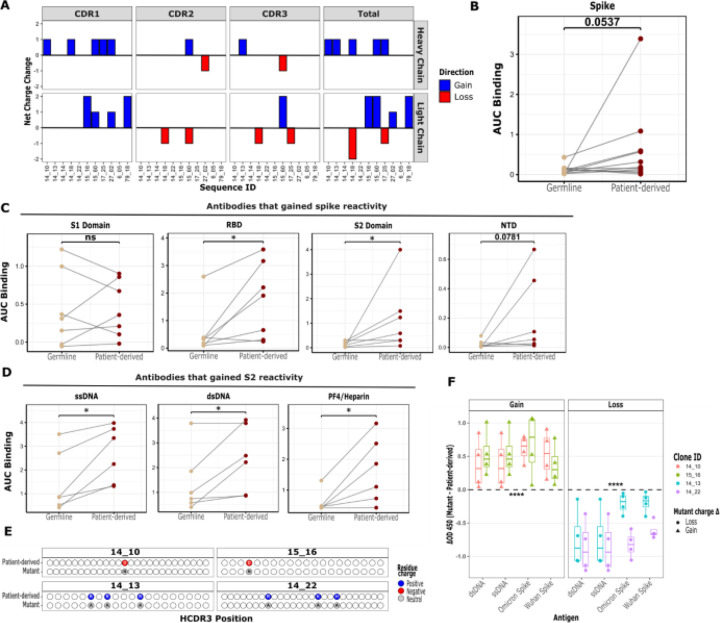
SARS-CoV-2 anti-spike reactivity is accompanied by gain of positive charge and increased autoreactivity. (**A**) Net charge changes across CDRs of polyreactive antibodies following somatic hypermutation. For each antibody, charge differences between patient-derived and inferred germline sequences are shown for heavy- and light-chain CDR1, CDR2, CDR3, and total CDR regions. Bars indicate gain (blue) or loss (red) of each region. (**B**) Pairwise comparison of spike binding by germline-reverted and patient-derived polyreactive antibodies measured by ELISA. Area under the curve (AUC) values are shown for individual antibodies (n = 19). Lines match germline and patient-derived pairs. Statistical significance was assessed using a paired Wilcoxon signed-rank test. (**C**) Antiviral pairwise comparison of germline versus patient-derived antibodies that gained spike reactivity (n = 7). ELISA AUC values are shown for spike subunits: S1 domain, receptor binding domain (RBD), S2 domain, and the N-terminal domain (NTD). (**D**) Autoreactivity pairwise comparison of germline versus patient-derived antibodies that gained S2 domain reactivity (n = 6). ELISA AUC values are shown for autoantigens: single stranded DNA (ssDNA), double stranded DNA (dsDNA), and platelet factor-4(PF4)/Heparin complexes. Statistical significance was assessed using a paired Wilcoxon signed-rank test. (**E**) Mutation schematic depicting amino acid changes from patient-derived heavy-chain CDR3 (HCDR3) sequences to mutant HCDR3 sequences for each antibody. Blue indicates positive, red indicates negative, and gray indicates neutral amino acids. (**F**) Mutation-induced changes in binding (ΔOD450; mutant – patient-derived) were measured across dsDNA, ssDNA, and two variants of spike (Omicron and Wuhan) and four concentrations (100, 33, 11, and 3.47ug/mL). Each point indicates each concentration difference, shape and box location indicate gain or loss of charge in the mutant, and color indicate antibody clone identity. Significance was determined by one-sided Wilcoxon signed-rank tests relative to zero. *p < 0.05, ****p < 0.0001.

To determine whether charge-associated polyreactivity arises stochastically or is acquired during antiviral responses, we generated germline-reverted versions of the polyreactive antibodies. Comparison of net charge changes across the CDRs of both heavy and light chains revealed that 10/11 patient-derived polyreactive antibodies acquired positive charge or lost negative charge, resulting in a net increase in charge across multiple CDRs relative to the inferred germline sequences (Fig. 5A). Only a single clone (14_18) exhibited an overall loss of charge (Fig. 5A). We next asked whether these charge changes were associated with altered antiviral reactivity. AUC analysis of binding curves comparing germline and patient-derived antibodies showed a strong trend toward an increase in spike binding following somatic mutation (Fig. 5B and Supplemental Fig. 3A), suggesting that affinity maturation toward spike is associated with charge gain within the CDRs. To assess whether enhanced spike binding was driven by a particular subunit of spike, we next tested whether the germline and patient-derived clones that gained spike reactivity against various spike subunits (S1 domain, S2 domain, RBD, and the S1 N-terminal domain (NTD)). Antibodies that gained spike reactivity in patient-derived clones showed a significant increase in the S2 domain and RBD binding; meanwhile, there was a trend toward an increase in the NTD (Fig. 5C and Supplemental Fig. 3, B-D). We next assessed whether antibodies that increased S2 reactivity also acquired higher levels of autoreactivity. Indeed, all the antibodies that gained S2 reactivity also exhibited significant increases in binding to autoantigens, including dsDNA, ssDNA, and PF4/heparin complexes (Fig. 5D and Supplemental Fig. 3E). Together, these data indicate anti-S2 responses are accompanied by a gain of positive charge within antibody paratopes that also enhance autoreactivity.

To definitively demonstrate positive plays a critical role in the recognition of spike and autoantigens, we generated 2 pairs of charge mutants from antibodies that exhibited polyreactivity and had similar HCDR3 lengths. We generated antibody mutants that lost positive charge (14_13 and 14_22) or negative charge (14_10 and 15_16) (Fig. 5E). Strikingly, loss of overall positivity within the HCDR3 decreased binding at all titration concentrations to dsDNA, ssDNA, Wuhan spike, and a more recent spike variant, Omicron, whereas loss of negative charge increased binding to these antigens (Fig. 5F). Together, these data demonstrate that positive charge plays a critical role in the ability of these polyreactive antibodies to recognize both autoantigens and spike.

### Positively charged antibodies preferentially target the negatively charged Wuhan but not the positively charged Omicron spike.

**Figure 6:**
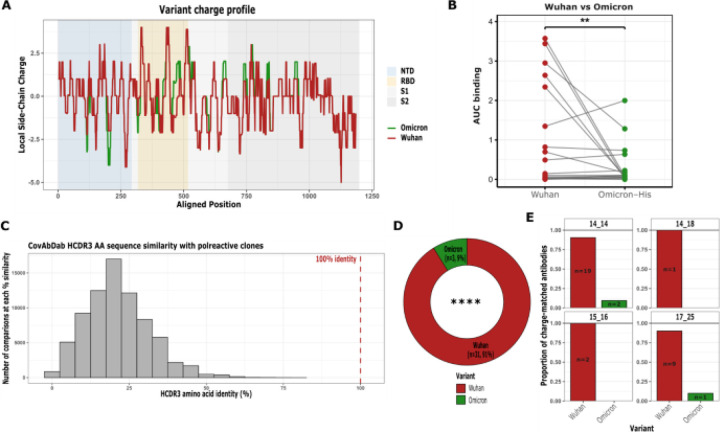
Spike variant surface properties are associated with distinct charge biases in the responding antibody repertoire. (**A**) Comparison of predicted local net charge across aligned SARS-CoV-2 spike protein sequences from the Wuhan and the B.1.1.529 (Omicron) variants utilized in the ELISA assays. Local net charge was calculated along the spike sequence and plotted by aligned position, with domain boundaries (NTD, RBD, S1, and S2) indicated by color. (**B**) Pairwise comparison of polyreactive antibody binding to Wuhan and Omicron spike proteins measured by ELISA AUC (n = 19). Area under the curve (AUC) values are shown for individual antibodies. Lines connect paired measurements for each clone. Statistical significance was assessed using a paired Wilcoxon signed-rank test. (**C**) CoVAbDab antibody heavy-chain CDR3 (HCDR3) sequence similarities (n = 12,793) compared to the 11 polyreactive antibody HCDR3 sequences. The dotted line represents 100% sequence similarity. (**D**) Charge-matched CoVAbDab antibodies to the 11 polyreactive antibodies preferentially recognize ancestral SARS-CoV-2 spike. HCDR3 region amino acid layouts from the 11 polyreactive antibodies were compared to antibodies in the CoVAbDab database to identify those with identical charge distributions. The donut plot shows the distribution of charge-matched antibodies and the respective variant reactivities. Wuhan binding antibodies comprised 31 of 34 (91.2%). Statistics were assessed using a one-sided exact binomial test. (**E**) Variant binding distributions for individual clones within charge-matched CoVAbDab antibodies. The y-axis shows the spike variant binding fraction of CoVAbDab matched antibodies to their polyreactive clone charge-match.

Previous work has shown that the SARS-CoV-2 spike protein has progressively acquired an increased net surface charge since its initial emergence^20^. To see how the charge of the virus may influence antibody responses, we first quantified local and domain-level charge differences between the two spike proteins utilized in our ELISAs. Compared with the Wuhan spike, B.1.1.529 (Omicron) exhibited increased net charge across multiple regions, including the RBD, S1, and S2 subunits, while exhibiting reduced charge in the S1-NTD (Fig. 6A, Table 2, and Supplemental Fig. 4, A and B), consistent with the previous report^20^. To determine whether spike variants with distinct charge properties elicit differentially charged antibody responses, we compared the binding of all 19 antiviral antibodies in our cohort against the Wuhan and Omicron spike proteins. ELISA analysis revealed a significant reduction in binding of all 19 patient-derived polyreactive antibodies to Omicron spike compared to Wuhan spike (Fig. 6B). Notably, the Omicron spike used in the assay contains a His-tag, whereas the Wuhan spike does not. To control for any effect of the His tag, we repeated the ELISA with a His-tagged Wuhan spike and found a similar reduction in binding to the Omicron spike (Supplemental Fig. 4C). Comparison of antibody binding to Wuhan spike with and without the His tag revealed only marginal differences in some antibodies, without a consistent pattern across clones, indicating that the His tag does not account for the reduced binding to Omicron spike. (Supplemental Fig. 4D).

Since our clone number likely represents a small subset of polyreactive antibodies from COVID-19 patients, we utilized The Coronavirus Antibody Database (CoVAbDab), an online database that compiles and retains SARS-CoV-2 antibody information from a large number of publications, to determine whether these antibodies can be identified at the population level^32^. To do this, we first subset the CoVAbDab to include only spike-reactive antibodies. Then, we compared the HCDR3 sequences of our polyreactive antibodies to those found in the CoVAbDab. Consistent with Fig. 3A, we observed little to no HCDR3 sequence convergence between our polyreactive antibodies and those in the CoVAbDab (Fig. 6C). The lack of sequence convergence is in line with previous reports of limited sequence convergence of polyreactive antibodies and further suggests previous work done on sorted high affinity antigen-specific antibodies may have limited access to polyreactive antibodies^26,33,34^. Because charge appears to be a critical determinant of autoreactivity of these antibodies, we next identified antibodies in the CoVAbDab with both identical HCDR3 length and charge distribution across the HCDR3 region, as these are likely to exhibit similar charge-based reactivity. Strikingly, 91% (n = 31) of the identified antibodies bound the Wuhan variant spike, while only 9% (n = 3) bound the Omicron strain (Fig. 6D), consistent with positively charged antibody paratopes favoring, the negatively charged, Wuhan variant binding. Additionally, the polyreactive clones with charge-matching CoVAbDab sequences (14_14, 14_18, 15_16, & 17_25) all exhibited high levels of autoreactivity to dsDNA, ssDNA, and PF4/Heparin based on ELISA data in Fig. 2B (Fig. 6E). These data indicate that charged antibodies respond differently to variably charged spike variants based on reciprocal charge and not sequence homology. Thus, more negatively charged spike variants may yield repertoires enriched for positively charged antibodies.

### Spike variant charge is associated with corresponding charge changes in the responding antibody repertoire.

**Figure 7:**
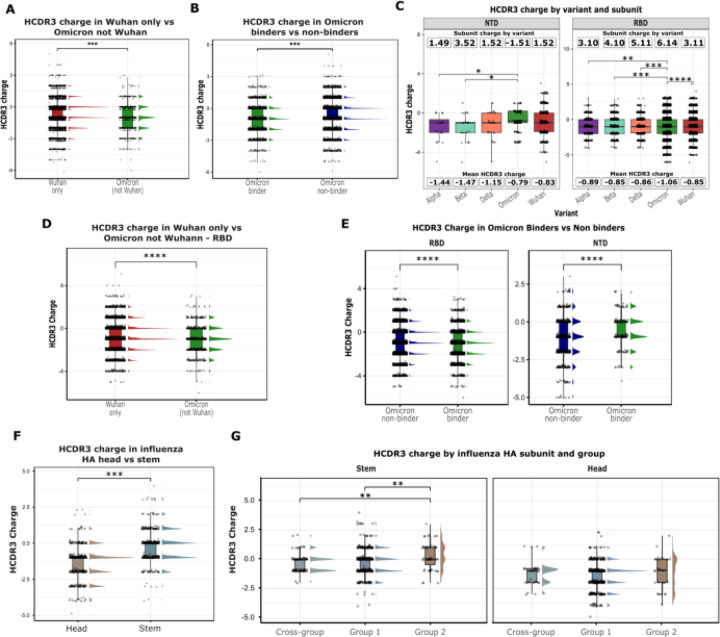
SARS-CoV-2 spike variant charge influences the corresponding antibody repertoire charge. (**A**) Distribution of heavy-chain CDR3 (HCDR3) charge among CoVAbDab antibodies that bind Wuhan spike exclusively (Wuhan only) (n = 7,273) versus antibodies that bind Omicron but not Wuhan (Omicron (not Wuhan)) (n = 758). Wuhan only antibodies had overall higher HCDR3 charge than those that bound Omicron but not Wuhan. Colored peaks parallel to boxplots indicate dot distribution for each group. (**B**) Comparison of HCDR3 charge between antibodies that bound Omicron (Omicron binder) (n = 3,009) and antibodies that bound other variants, but not Omicron (Omicron non-binder) (n = 9,788). Omicron-binding antibodies exhibit significantly lower HCDR3 charge compared with nonbinding antibodies. Colored peaks parallel to boxplots indicate dot distribution for each group. (**C**) HCDR3 charge comparison of antibodies that bound subunits of each SARS-CoV-2 variant. Top boxes indicate the charge of each subunit for each variant, and bottom boxes indicate the mean HCDR3 charge of the antibody repertoire (NTD n values left to right; 24, 27, 32, 145, 529; RBD n values left to right; 485, 650, 618, 27,225, 7,732). (**D**) Spike subunit breakdown of the HCDR3 antibodies responding to Wuhan only (n = 5,855) versus Omicron (not Wuhan) (n = 730), and (**E**) Omicron binder versus Omicron non-binder (RBD n values left to right; 6,245, 2,824; NTD n values left to right; 692, 167). Colored peaks parallel to boxplots indicate dot distribution for each group. (F) HCDR3 charges for influenza hemagglutinin (HA) binding antibodies, split by each subunit; head (n = 493) and stem (n = 498). (G) HCDR3 charges for antibodies binding HA subunits and split by influenza group reactivity. Cross-group indicate antibodies that bound both groups (Stem n values left to right; 81, 362, 55; Head n values left to right; 33, 430, 30). Statistical significance was assessed using a Wilcoxon rank-sum test. *p < 0.05, **p<0.01, ***p < 0.001, ****p < 0.0001.

Given that positively charged HDCR3s preferentially bound the Wuhan spike over the Omicron spike based on our polyreactive antibody and CoVAbDab comparisons, we next sought to determine whether charge-dependent differences in antibody reactivities to variants could be observed at the repertoire level. To address this, we utilized the CoVAbDab, with the known caveat that this database is enriched for early pandemic strains. We first examined whether the HDCR3s of antibodies reported to bind Wuhan spike exclusively differed from those that bound Omicron but not Wuhan. We observed a significant reduction in overall HCDR3 charge of those that bound Omicron but not Wuhan, compared with Wuhan-specific antibodies (Fig. 7A). Because the Omicron spike is one of the most positively charged variants, we then determined whether antibodies that bound Omicron without detectable binding to other variants show significantly lower HCDR3 charge. Consistently, the Omicron-reactive repertoire showed a significant reduction in overall HCDR3 charge compared with non-Omicron-reactive antibodies (Fig. 7B).

To further assess whether spike charge influences the charge of the responding repertoire, we examined whether variation in spike subunit charge across variants associated with HCDR3 charge changes. When we compared spike-binding antibodies in CoVAbDab, Omicron drove significant differences in responding HCDR3 repertoires for both the RBD and NTD compared to several other variants (Fig. 7C and Supplemental Fig. 5A). These data are consistent with Omicron’s RBD and NTD being the most electrostatically different than other variants. Consistently, comparisons of the epitopes between Wuhan only binders and Omicron, but not Wuhan, showed that the RBD, which is more positively charged in Omicron, elicited a significantly reduced HCDR3 charge in the responding repertoire (Fig. 7D and Supplemental Fig. 5B). This was also observed when we compared the Omicron non-binders and Omicron binders, where the Omicron non-binders’ antibodies had a more positively charged HCDR3 versus the Omicron binders in the RBD, and the opposite for the S1-NTD, consistent with the charge differences of each subunit (Fig. 7E and Supplemental Fig. 5C). Since the CoVAbDab is also enriched for RBD-specific antibodies, not all spike subunits had sufficient numbers for variant comparisons. These data show that the charge of the spike variant dictates the overall responding repertoire charge, even at the subunit level.

### Hemagglutinin charge in influenza variants and subunits correlates with reciprocally charged antibody repertoires in influenza patients and vaccinees.

To determine whether charge-based antiviral responses extend beyond COVID-19, we investigated whether influenza also elicits differentially charged repertoires. To do this, we utilized the influenza antibody and reactivity database generated by Wang et al from influenza patients and vaccinees^35^. The hemagglutinin (HA) of the influenza virus consists of two major subunits, the head and stem. Previous work has shown that the head of HA is more positively charged than the negatively charged stem, although this may fluctuate depending on the specific strain in question^36^. We first asked whether the positively charged head could elicit differentially charged repertoires compared to the negatively charged stem. Indeed, the head generated a more negatively charged repertoire than the stem (Fig. 7F). Of the two subunits of HA, the stem is more conserved, and the group 2 stem is more negatively charged than the group 1 stem^37^. Importantly, the repertoires elicited by the group 2 stem were more positively charged than those elicited by the group 1 stem or the cross-reactive antibodies (cross-group) (Fig. 7G). These findings are consistent with our observation that spike subunits elicit reciprocally charged repertoires based on subunit charge (Fig. 7C). In contrast, the highly variable HA head regions did not elicit charge differences in antibody repertoires among influenza groups (Fig. 7G). These data provide further evidence that differentially charged viral antigens elicit reciprocally charged antibody repertoires and suggest that charge-driven antiviral responses concomitant with polyreactivity may extend beyond COVID-19.

## Discussion

Severe viral infections are associated with immune dysregulation and autoantibody production, yet the mechanisms linking antiviral responses to autoreactivity remain unclear^4,8,18,38^. Here, by integrating antibody profiling, repertoire analysis, and viral evolution, we identify a novel charge-driven mechanism linking antiviral immunity to autoantibody development in severe COVID-19. Antibodies targeting the SARS-CoV-2 spike protein, particularly its charged surfaces (RBD and S2 domain), acquire autoreactivity and gain reactivity to autoantigens, such as dsDNA. Despite similar binding profiles, these antibodies lack sequence convergence, indicating that canonical selection is not the primary driver^24,25^. Instead, sequence analyses reveal enrichment of positively charged residues within HCDR3 as the dominant determinant, consistent with the intrinsic promiscuity of charge-enriched paratopes^25,26^. Notably, the polyreactive antibodies identified here retain antiviral binding, indicating that autoreactivity in COVID-19 emerges during ongoing antiviral responses, a concept challenging current dogma that charge is eliminated during affinity maturation^39,40^. Importantly, those that gained S2 and RBD binding also gained reactivity for autoantigens, including dsDNA, ssDNA, and PF4/Heparin. Further, mutagenesis showed loss of positivity in the HCDR3 of the polyreactive antibodies directly reduced binding to both viral and autoantigens. Together, these findings support a model in which charge interactions, rather than structural mimicry, govern antigen recognition. Thus, our study identifies a novel mechanism by which autoreactivity emerged independent of structural homology. This framework contrasts current paradigms that emphasize structural convergence or loss of HCDR3 charge during affinity maturation^39,40^, and instead suggests that autoreactivity can arise directly from affinity maturation.

This model may help explain adverse events associated with SARS-CoV-2 vaccination, including thrombosis and thrombocytopenia syndromes (TTS) following adenovirus vector–based vaccines (VITT), and more rarely, mRNA-based vaccines^41,42^. Although VITT has been proposed to arise through molecular mimicry between the adenoviral nucleoprotein pVII and PF4^43^, electrostatic interactions likely contribute to the generation of these cross-reactive antibodies. Such a mechanism resembles the charge-driven affinity maturation associated with autoreactivity in this study and may help explain why autoreactive responses have been observed following other SARS-CoV-2 vaccines, where the underlying mechanisms remain poorly understood^42,44^. Consistent with this model, the SARS-CoV-2 spike protein has increased in positive charge since 2019^45,46^, a phenomenon that may be explained through charged antibody-driven selection for a more positively charged spike protein. Consistent with this model, positively charged antibodies that are reactive to the Wuhan strain show reduced reactivity to Omicron, and Omicron-binding repertoires exhibit decreased HCDR3 charge, indicating that spike variant charge shapes antibody responses. Although our data does not demonstrate direct selection of autoreactive antibodies based on variant charge, they suggest that viral evolution influences the responding repertoire through charge, with increased potential for autoreactivity in responses that elicit positively charged repertoires. Further, our influenza analysis demonstrated similar antibody repertoire responses based on the charge of the subunit and variant.

Consistent with the autoreactivity we observed with the anti-S2 antibodies, recent work has demonstrated antibodies with reactivity toward the negatively charged stem exhibited more autoreactivity than those with reactivity to the positively charged HA head^47^. Additionally, the group 2 elicited a positively charged repertoire, which contains the HA variant H3. Monoclonal antibodies from influenza patients that are specific for H3 have been shown to be ANA and other autoantibodies in humans^18^. H3 vaccination has also been shown to elicit autoantibodies in mice, consistent with our model that charged antigens can influence vaccine reponses^18^. Therefore, this novel concept may have very broad implications for immunogens and vaccines that wish to avoid generation of positively charged repertoires that inherently increase odds of autoreactivity. Consistent with this, vaccines that have used the Wuhan strain in vaccine design have observed autoreactive responses following vaccination^42,44^.

This study has several limitations. We cannot fully exclude a contribution from bystander activation in the development of these antibodies. The polyreactive antibodies we identified may have been developed previously in autoreactive anergic B cells that have been activated due to high levels of inflammation. Although this is possible, it is unlikely since the majority of our antibodies came from differentiated B cell subsets, which are unlikely to be anergic. In addition, the monoclonal antibody cohort was enriched for the RKH/Y5 motif, potentially biasing toward positively charged antibodies. Although this is true, we demonstrated that at the repertoire level, spike charge plays a significant role in the responding antibody repertoire charge. Additionally, clonal evolution within the HCDR3 remains difficult to infer, as germline reversion cannot reliably capture changes in this region. As a result, although we observe charge gain across multiple CDRs, precise determination of charge changes within the HCDR3 itself remains limited. Finally, because ANA antibodies are relatively common in the general population, their pathogenicity in the context of COVID-19 requires further *in vivo* investigation.

Taken together, our findings support a model in which autoreactive antibodies in severe COVID-19 arise as a consequence of charge-mediated antiviral responses. Somatic mutation toward SARS-CoV-2 spike increases paratope charge, enhancing binding to charged viral epitopes while concurrently expanding reactivity to autoantigens. Thus, our study provides critical evidence suggesting that charge of antigens needs to be considered when developing vaccines and immunogens to prevent the development of autoimmunity-prone repertoires.

## Materials and Methods

### Sex as a biological variable

Both male and female patients were utilized in this study. Samples were not chosen based on sex, as sex was not considered as a biological variable in this study.

### Study design

The objective of this study was to determine the origin of anti-nuclear antibodies in COVID-19. This study utilized samples from a previously established cohort of hospitalized COVID-19 patients, including plasma for serologic analyses (n = 76) and PBMCs for single-cell RNA sequencing and paired B cell receptor (BCR) profiling (n = 11). Experimental units included patient plasma, single B cells, and recombinant monoclonal antibodies generated from paired heavy- and light-chain sequences selected the criteria described in Antibody selection. Sample size was determined by cohort availability, and no prospective power calculations or stopping rules were applied. Inclusion criteria required confirmed SARS-CoV-2 infection and availability of baseline samples. No data were excluded except in cases of technical failure. Experiments were performed with technical replicates. Subjects were not randomized, and no blinding was applied. Both males and females were utilized in this study.

### Patients and sample collection

11 COVID-19 patients who were hospitalized at Froedtert Hospital were entered into the open-label clinical trial Evaluating the Efficacy and Safety of High-Titer Anti-SARS-CoV2 plasma in hospitalized patients with COVID-19 infection (NCT04354831). All patients were confirmed COVID-19 positive via RT-PCR testing. Blood was drawn from patients prior to the administration of any intervention. All samples used in this study were collected upon admittance into the hospital, denoted as day 0. PBMC isolation, cryopreservation, storage conditions, and procedures were performed as reported in Witman *et al*.^22^. Briefly, whole blood was processed by density gradient centrifugation, cryopreserved in serum-containing freezing media, and stored in liquid nitrogen until use. All analyses presented here were performed on samples from this previously described cohort and address biological questions distinct from those examined in the original study. Non-COVID-19 patients with acute respiratory symptoms (ARS) were acquired from the Zuckerberg San Francisco Hospital.

### PBMC Isolation and cryopreservation

Cryopreserved PBMCs were thawed, stained, sorted, and processed for single-cell RNA sequencing and paired B cell receptor sequencing using the 10x Genomics Chromium 5′ platform following the same experimental workflows and reagent versions reported previously^22^. Library preparation, sequencing, and initial data generation were conducted as described^22^.

### Single-cell RNA-sequencing and V(D)J analysis

Gene expression data were processed and analyzed using the same computational pipeline previously established^22^, including quality control filtering, normalization, batch correction, dimensionality reduction, clustering, and cell type annotation. Immunoglobulin-associated genes were regressed prior to normalization. All parameters and thresholds matched those used in the prior study unless otherwise noted. Single-cell V(D)J sequencing data were processed, and clonotypes were assigned using the identical alignment, filtering, and clonal inference pipeline described previously^22^. Immunoglobulin sequence annotations and clonal relationships were integrated with matched transcriptomic profiles for downstream analyses. Mutation frequencies for heavy- and light-chain variable regions were determined utilizing the SHazaM package, and lineage trees were assessed using the package Alakazam from the Immcantation group^48^. Sequencing files are available in the NCBI BioProject database under accession number PRJNA1391649.

### Antibody selection and expression

Monoclonal antibodies were selected for recombinant expression based on the presence of an RKH/Y5 motif, which is either 3 cationic residues (R, K, H) or 5 tyrosine residues (YYYYY), or clonal expansion within single-cell datasets (> 3 expanded cells). The exact definition of RKH/Y5 motifs has been described in a previous publication^23^. Antibodies that belonged to clonally expanded B cells were limited to the single clone most distant from germline-inferred sequences, with a preference for IgG over IgM. Selected heavy- and light-chain V(D)J sequences were annotated using IMGT and cloned into human IgG1 heavy-chain and kappa or lambda light-chain expression vectors by a commercial vendor.

### Antibody production and purification

V(D)J sequences from V(D)J sequencing were compiled and loaded into IMGT to generate antibody sequences. Recombinant antibody vectors were generated utilizing GeneArt Synthesis cloning. GeneArt recombinant antibodies were produced by transient co-transfection of Expi293F cells with paired heavy- and light-chain expression vectors using a commercial transfection system (Gibco). Culture supernatants were harvested after 5 days, and IgG was purified by Protein A affinity chromatography, buffer-exchanged, and quantified prior to downstream analyses.

#### Antinuclear antibody (ANA) ELISA and immunofluorescence:

The ANA ELISA (Werfen) was conducted according to Werfen’s QUANTA-LITE protocol. In brief, patient plasma was diluted 1:41, detected using an anti-human HRP conjugated secondary, and read at 450nm-570nm. Sample ANA scores were determined as (Sample OD/ANA ELISA Low Positive OD) × ANA ELISA Low Positive (units). Samples with > 20 units were considered moderate, and samples with > 60 units were considered strong positives. ANA immunofluorescence was conducted using Werfen NOVA Lite Hep-2 ANA kit. Samples were prepared by kit instructions, and slides were imaged using the Nikon Ti2 Widefield inverted microscope at 40x. Staining patterns were assessed and categorized according to the CDC's definition of ANA staining patterns^21^.

#### Polyreactivity and RBD ELISAs:

LPS, dsDNA (Sigma Aldrich), and ssDNA were diluted to 10 μg/mL. PF4 and Heparin were diluted to 10ug/mL and 0.4U/mL, respectively. RBD-His (RBD) was diluted to 100ng/mL. All antigens were plated overnight at room temperature. Plates were washed 3x with 0.1% PBS-Tween, then blocked with 3% BSA in PBST for 1 hour. Serum was diluted 1:100, and purified IgG was diluted to 10 μg/mL, plated in a volume of 50 μL, and incubated for 1 hour. Plates were washed using 3% BSA PBST, and anti-human IgG HRP-conjugated (Southern Biotech) was diluted 1:5000, and 100 μL was added to each well. Plates were washed, and equal parts of substrate A & B (Seracare) were added to each well and incubated for 20 minutes. Reactions were stopped by adding 25 μL of 1N H_2_SO_4,_ and plates were read at 450nm and corrected by subtracting 570nm.

#### Spike subunit ELISAs:

Recombinant SARS-CoV-2 spike subunits (full-length spike, and His-tagged S1, S2, and NTD; R&D systems) were plated at 2 ng/uL overnight at room temperature. Plates were washed three times with 0.1% PBS-Tween (PBST) and blocked with 5% milk in PBST for 1 hour. Purified IgG was diluted to 100, 33, 11, and 3.47 μg/mL, added at 50 μL per well in duplicate, and incubated for 1 hour. Plates were washed with PBST, and anti-human IgG HRP-conjugated secondary antibody (Southern Biotech) was diluted 1:5000 in 1% milk in PBST and added at 50 μL per well. Plates were washed, and equal parts of substrate A and B (Seracare) were added to each well and incubated for 20 min. Reactions were stopped with 25 μL of 1 N H_2_SO_4_, and absorbance was measured at 450 nm with background correction at 570 nm.

### Antibody paratope analysis and clustering

VDJ sequences were processed and submitted to IMGT/High V-Quest for proper sequence alignment^49^. Amino acid sequences were then checked for productivity, and unproductive sequences were removed. Productive amino acid sequences were run utilizing AbMAP in Python^28^. Sequence embeddings were generated using AbMAP with the Bepler–Berger protein language model (embed_type = “beplerberger”) without mutation-based augmentation (num_mutations = 0). Embeddings aggregated to sequence-level representations by mean pooling across positions, resulting in fixed-length feature vectors for each antibody. Dimensionality reduction was performed using principal component analysis (PCA), followed by uniform manifold approximation and projection (UMAP) using the top principal components. UMAP visualizations were generated to compare the distribution of polyreactive, vaccinee, and convalescent antibodies within the embedding space. To quantify structural convergence, pairwise cosine similarity and nearest-neighbor distances were computed within each group using the embedding vectors. Nearest-neighbor distance was defined as the Euclidean distance to the closest sequence within the same group, excluding self-comparisons. Differences between groups were assessed using Wilcoxon rank-sum tests with Benjamini–Hochberg.

#### Antibody docking and interface analysis:

Fab structures were predicted using IgFold with PyRosetta-based refinement enabled^50^. Predicted Fabs were docked onto the SARS-CoV-2 spike ectodomain (PDB: 6VXX) using ClusPro in antibody mode, with all non-CDR loops masked to direct sampling toward the interface^51^. The top-ranked docked complex from each run was carried forward for structural relaxation. Relaxation was performed using GROMACS 2024.2 with the AMBER99SB-ILDN force field and explicit TIP3P solvent in a cubic periodic box with a minimum 1.0 nm solute-to-wall distance^52^. The system was neutralized and brought to 150 mM NaCl prior to a steepest-descent energy minimization, followed by NVT and NPT equilibration. A production MD run was then performed with GPU-accelerated nonbonded interactions. The final frame of the production trajectory was extracted as the relaxed complex structure. Interface analysis was carried out by uploading the relaxed complex PDB to PDBePISA to determine buried surface area, interfacial residue contacts, and predicted interface solvation energies^53^.

### Antibody germline reconstruction

VDJ sequences from B cells were reverted to germline sequences by utilizing ChangeO. Gaps in sequences were removed, and the subsequent fragments were combined. Unidentifiable N nucleotides were matched to the VDJ sequence isolated from patient-derived B cells to generate complete sequences. Germline reverted sequences were then loaded into IMGT/V-Quest to confirm amino acid sequences and productive reconstruction.

### Spike and spike subunit charge calculations

Predicted net charge of spike proteins was calculated using a pH-dependent model that accounts for the protonation states of ionizable residues. For each amino acid, side-chain charge was estimated using the Henderson–Hasselbalch equation with standard pKa values (Asp, 3.9; Glu, 4.1; His, 6.0; Cys, 8.3; Tyr, 10.1; Lys, 10.5; Arg, 12.5). The contributions of the N- and C-termini (pKa 9.69 and 2.34, respectively) were included for full-length sequences. Net charge at physiological pH (pH 7.4) was calculated as the sum of all residue-specific charges. For domain-level analyses, charge was computed over defined spike subunits (NTD, RBD, S1, and S2) based on Wuhan reference coordinates. For local electrostatic profiling, a sliding-window approach (window size = 15 residues) was used to approximate regional charge environments across aligned Spike sequences, reflecting the scale of antibody–epitope interactions. For variant comparisons, spike sequences were generated by introducing lineage-defining mutations into the Wuhan reference sequence, and charge was calculated identically for each variant.

### Antibody heavy-chain CDR3 (HCDR3) charge calculation

HCDR3 charge was quantified using a length-normalized weighted charge metric. Amino acids were assigned charge values of + 1 (K, R), + 0.1 (H), and − 1 (D, E), while all other residues were treated as neutral. The total charge was summed across the sequence and divided by the total number of residues to account for differences in CDR3 length.

### CoVAbDab sequence processing

Published SARS-CoV-2 spike-binding antibody sequences were retrieved from the CoVAbDab database and filtered to entries targeting the spike protein^32^. Net charge at physiological pH was calculated for the heavy-chain variable domain, light-chain variable domain, HCDR3, and LCDR3 of each antibody by summing residue-level contributions, assigning + 1 to lysine and arginine, − 1 to aspartate and glutamate, and + 0.1 to histidine. Variant reactivities were extracted from the CoVAbDab metadata to classify antibodies by the variant binding.

### Statistical analysis

Statistical analyses were performed using R (version 4.4.1). A two-sided p value less than 0.05 was considered statistically significant. Comparisons between two independent groups were performed using Wilcoxon rank-sum tests, and paired comparisons were assessed using Wilcoxon signed-rank tests. Categorical data were analyzed using Fisher’s exact test. Proportions were evaluated using exact binomial tests where appropriate. For analyses involving multiple comparisons, p values were adjusted using the Benjamini–Hochberg false discovery rate correction where applicable. ELISA-based comparisons were performed on area under the curve (AUC) values derived from serial dilutions. Correlation analyses were performed using Pearson correlation unless otherwise specified. No samples were excluded except in cases of technical failure. Outliers were not excluded unless explicitly stated. Sample sizes, statistical tests, and exact p values are indicated in the figure legends.

## Supplementary Material

Supplementary Files

This is a list of supplementary files associated with this preprint. Click to download.

- PolyreactivePaperSuppFigures.docx

## Figures and Tables

**Table 1:** Quantitative summary of predicted interface properties derived from PDBePISA analysis for (27_02, 15_60, and 14_22) and spike. Values represent compiled predicted interface information for both heavy and light chains to the crystallized spike trimer (RCSB Protein Data Bank; 6VXX).

Metric	Heavy-Chain			Light-Chain		
Clone ID	27_02	15_60	14_22	27_02	15_60	14_22
Interface Area (Å)	816.5	793.2	884.3	190.3	249.3	38.6
Total # hydrogen bonds	9	9	12	1	0	1
Total # salt bridges	8	6	13	0	0	0

**Table 2:** Net charge calculation of spike subunit charges and total spike charges in Wuhan and Omicron spike variants. Charges were calculated based on the assumed amino acid charge at a pH of 7.

	Wuhan	Omicron
Net charge NTD	1.52	−1.51
Net charge RBD	3.11	6.14
Net charge S1	3.03	6.03
Net charge S2	−13.98	−9.94
Net charge of spike (S1 + S2)	−10.95	−3.91

## Data Availability

The scRNA-seq and paired V(D)J-seq datasets generated in this study have been deposited in the NCBI BioProject database under accession number PRJNA1391649. Supporting analytic code used for data processing and analysis is available on CodeOcean. Supporting data files and raw data used to generate figures can be found on figshare at https://figshare.com/s/799247e3fb51e25dba3a. CoVAbDab data for repertoire analysis can be found at https://opig.stats.ox.ac.uk/webapps/covabdab/. Convalescent and vaccinee RBD-specific antibody sequences were take from https://doi.org/10.1038/s41586-020-2456-9 and https://doi.org/10.1038/s41586-022-05609-w, respectively. Influenza antibody information can be found at https://doi.org/10.1016/j.immuni.2024.07.022.
